# Demonstration of bioplastic production from CO_2_ and formate using the reductive glycine pathway in *E. coli*

**DOI:** 10.1371/journal.pone.0327512

**Published:** 2025-07-22

**Authors:** Daria Fedorova, Roee Ben-Nissan, Eliya Milshtein, Cassandra Reyes, Ghil Jona, Nili Dezorella, Gil Feiguelman, Rostislav Fedorov, Aya Gomaa, Ariel B. Lindner, Elad Noor, Ron Milo

**Affiliations:** 1 Department of Plant and Environmental Sciences, Weizmann Institute of Science, Rehovot, Israel; 2 Université Paris Cité, INSERM U1284, Center for Research and Interdisciplinarity (CRI), Paris, France; 3 The California Institute for Quantitative Biosciences, University of California Berkeley, Berkeley, California, United States of America; 4 Department of Life Sciences Core Facilities, Weizmann Institute of Science, Rehovot, Israel; 5 Electron Microscopy Unit, Weizmann Institute of Science, Rehovot, Israel; 6 Heidelberg Institute for Theoretical Studies, Heidelberg, Germany; Karl-Franzens-Universitat Graz, AUSTRIA

## Abstract

There is a strong need to develop technologies that reduce anthropogenic pollution and the dependence on nonrenewable Earth resources. One way of doing so is by harnessing biological systems for replacing the production of fossil-fuel based goods with low-environmental-impact alternatives. Recently, progress was made in engineering the model organism *E. coli* to grow using CO_2_ and formate as its only carbon and energy sources using the reductive glycine pathway (rGlyP). Here, we use this engineered strain of *E. coli* as a host system for the production of polyhydroxybutyrate (PHB), a biologically derived and biodegradable plastic. The production of PHB in this strain was confirmed using Nile red fluorescence microscopy, transmission electron microscopy, and HPLC analysis, with a yield of 0.172 ± 0.005 mg/L of PHB after 120 hours of incubation. Since formate can be efficiently generated from CO_2_ by electrochemical reduction using renewable energy sources, this study serves as a proof of concept for the emerging field of electro-bioproduction.

## Introduction

Currently, a large fraction of industrial chemicals are produced from petroleum and natural gas. These petrochemicals (such as ethylene and acetylene) are used as a feedstock for the production of plastics, fuel, textiles, cosmetics and fertilizers [1]. Using petroleum as a feedstock poses major problems and thus alternatives based on renewable feedstocks such as maize, sugar cane, or other plant-based sources, have been gaining momentum in recent years [2]. However, these bioproducts have a large land and water footprint and often compete with food production [3]. This could have consequences for food security, especially in the context of a growing population and projected yield reductions from climate change. Moreover, expanding cropland has a negative effect on biodiversity due to loss and fragmentation of natural habitats [4–7]. There is therefore a need to find a different alternative to bioproduction of chemicals which is scalable, efficient, and decoupled from human food production.

### Poly-β-hydroxybutyrate (PHB) production in microorganisms

**Fig 1:**
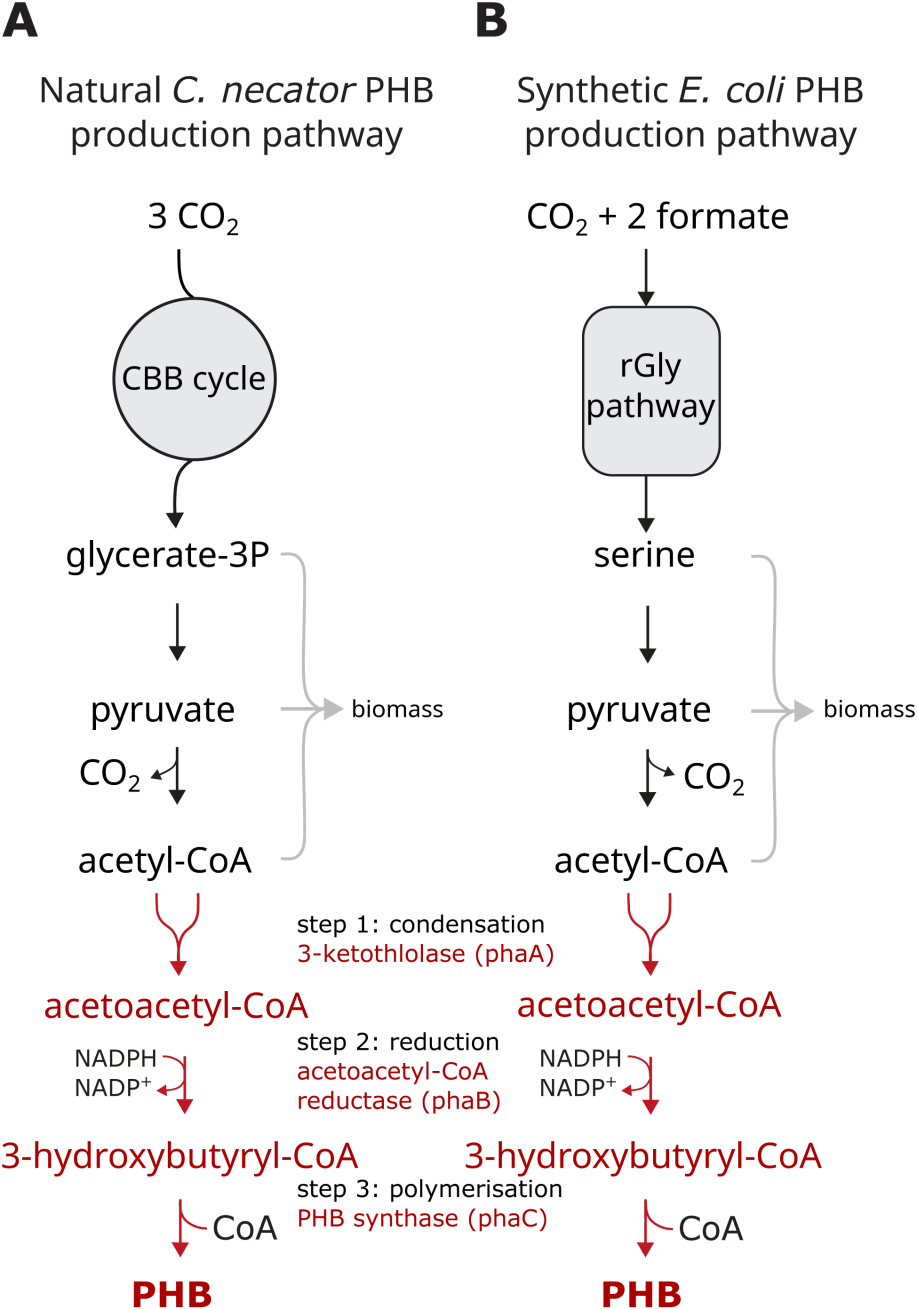
Metabolic pathway of PHB production in *C. necator* and rGlyP *E. coli.* ***(A)*** *Metabolic pathway of PHB production in C. necator using the Calvin-Benson-Bassham (CBB) cycle for carbon assimilation. The PHB pathway is highlighted in red.* ***(B)*** *Metabolic design of rGlyP E. coli producing PHB. Formate and CO*_*2*_ *are assimilated into carbon metabolism using the reductive Glycine pathway. PHB is produced from Acetyl-CoA by heterologous expression of the PHB operon (in red).*

One important class of industrial materials targeted for sustainable bioproduction is bioplastics, which aim to replace petroleum-derived plastics while minimizing environmental impact. Poly-β-hydroxybutyrate (PHB) is a naturally biodegradable polyester, synthesized natively in various microorganisms as a store of energy and carbon under nutrient-depleted conditions. Humanity mainly uses PHB for food packaging, as well as biocompatible and biodegradable implants, medical scaffolds, and encapsulation of medicines [8–12]. PHB can be produced in *E. coli* heterologously by expression of a PHB operon. As shown in Fig 1, PHB production in *E. coli* requires expression of three non-native enzymes: PhaA (3-ketothiolase), PhaB (acetoacetyl-CoA reductase) and PhaC (PHB synthase). PhaA condenses two molecules of acetyl-CoA to form acetoacetyl-CoA, which is then reduced by PhaB to create (R)-3-hydroxybutyl-CoA (3HB). PhaC then uses 3HB as the monomer for polymerising poly-3-hydroxybutyrate (PHB). On an industrial scale, PHB is produced heterotrophically in fermenters by bacteria such as *Cupriavidus necator* or recombinant *Escherichia coli*, using glucose as a feedstock [13]. *C. necator* is also capable of producing PHB natively from CO_2_ as the sole carbon source (using aerobic respiration of H_2_ as the energy source) [14,15]. However, unlike *E. coli*, *C. necator* has a poorly characterized genome and limited genome manipulation tools. This hinders the use of *C. necator* for producing non-native chemicals. Beyond organism-specific challenges, widespread adoption of PHB itself has been hampered by high production costs, driven largely by the use of expensive sugar-based substrates and suboptimal yields [16,17]. To address these challenges, research efforts have focused on strategies such as metabolic pathway optimization, process engineering, and exploring alternative carbon sources. C1 compounds have emerged as potentially more sustainable alternatives due to their lower environmental footprint compared to sugar-based substrates. However, their practical implementation currently faces significant technological and economic hurdles, which we hope future advancements will overcome. Here, we aimed to demonstrate a synthetically engineered C1-feeding *E. coli* as a platform for PHB bioproduction. This serves as a proof-of-concept for sustainable microbial production of other valuable biochemicals, assuming their biosynthetic pathways can be readily introduced into this well-characterized model organism.

### Engineering *E. coli* to feed on C1 compounds

Recently, progress has been made in engineering *E. coli* to feed on C1 compounds [18–21]. As a background to our work using one such *E. coli* strain, we highlight three studies that demonstrated growth on C1 feedstocks using the reductive glycine pathway (rGlyP), the reductive pentose phosphate (rPP) cycle, and the ribulose monophosphate (RuMP) cycle.

Kim and collaborators [18] introduced the reductive glycine pathway (rGlyP), an efficient route for direct formate and CO_2_ assimilation into central metabolism (Fig 1, left). By combining rGlyP with laboratory evolution approaches, they successfully achieved formatotrophic growth of synthetically engineered *E. coli*. This strain had a doubling time of less than 8 hours and a biomass yield of 2.3 gCDW per mol of formate. This growth was strongly coupled to formate as alternative electron donors are not effective with rGlyP. Additionally, this strain lacks a CO_2_ concentrating mechanism, requiring artificially high concentrations of CO_2_ for biomass accumulation.

Gleizer and collaborators [19] established complete synthetic autotrophy in *E. coli* by combining rational design and adaptive laboratory evolution. The evolved strain uses the reductive pentose phosphate cycle (rPP) cycle for all its biomass production and relies solely on CO_2_ and formate as its carbon and energy sources respectively. This strain displays a doubling time of 18 ± 4 hours and has a formate-to-biomass conversion yield of 2.8 ± 0.8 gCDW/mol formate. Further engineering of this strain may enable growth at ambient CO_2_ levels, which is useful in industrial settings. Furthermore, since carbon fixation was decoupled from the energy supply in this strain, the system is modular and can be readily adapted for the use of other electron donors (*e.g.,* methanol, hydrogen).

In another study, Keller and collaborators [21] transformed *E. coli* into a synthetic methylotroph that assimilates methanol via the energy-efficient ribulose monophosphate (RuMP) cycle. Methylotrophy was achieved after evolving a methanol-dependent *E. coli* strain over 250 generations in a continuous chemostat culture, using methanol as the sole source of carbon and energy. This strain is strongly coupled to methanol and cannot use any other energy source. Recently, this strain was engineered to demonstrate the production of four diverse biochemicals, one of which is PHB [22].

In light of the studies described above, we opted to incorporate a PHB production pathway from *C. necator* into the *E. coli* strain that was previously equipped with rGlyP [18]. While the strain that utilizes the RuMP cycle for growth on methanol supports relatively rapid growth, our aim was to showcase the production of PHB from formate, as it corresponds with the concept of a formate economy (as elaborated in the discussion section). Although both the autotrophic strain and the rGlyP strain are suitable for this purpose, the latter exhibited a significantly faster growth rate and is more amenable to straightforward genetic engineering

## Results

*C. necator* H16 naturally produces polyhydroxybutyrate (PHB) from acetyl-CoA as a carbon storage polymer that accumulates as granules in the cytoplasm. The three genes required for PHB production are located within a single operon. We set out to introduce the PHB production pathway into formate-utilizing strains of *E. coli* that rely solely on formate and CO_2_ as their sources of carbon and energy by using the reductive glycine pathway (rGlyP). rGlyP is a synthetic pathway that offers an efficient route for the direct assimilation of formate and CO_2_ into the rest of cellular metabolism (Fig 1B). A variant of this pathway was identified in the anaerobic bacterium *Desulfovibrio desulfuricans* [23]. Building upon previous work by Kim and collaborators [18], who successfully achieved formatotrophic growth in the engineered *E. coli*, we sought to adapt this strain for production of PHB. We validated that this strain grows quickly on rich media, making it amenable to fast genetic manipulation. Furthermore, its doubling time on M9 minimal media, supplemented with formate and CO_2_ as the only reducing and carbon sources, was less than 8 hours, sufficiently fast for our experimental setup. To produce PHB in *E. coli*, we introduced a pHB-4 plasmid [24] containing the PHB operon under the control of the auto-inducible promoter PthrC3 [24]. This promoter was selected due to its advantages over traditional inducible promoters, such as pT7 [25]. PthrC3 is an endogenous, short *E. coli* promoter that can auto-induce during early exponential growth, thereby enhancing efficiency and cost-effectiveness by eliminating the need for inducer chemicals in the media, which could also potentially be used as a carbon source (e.g., in Arabinose-inducible systems).

**Fig 2:**
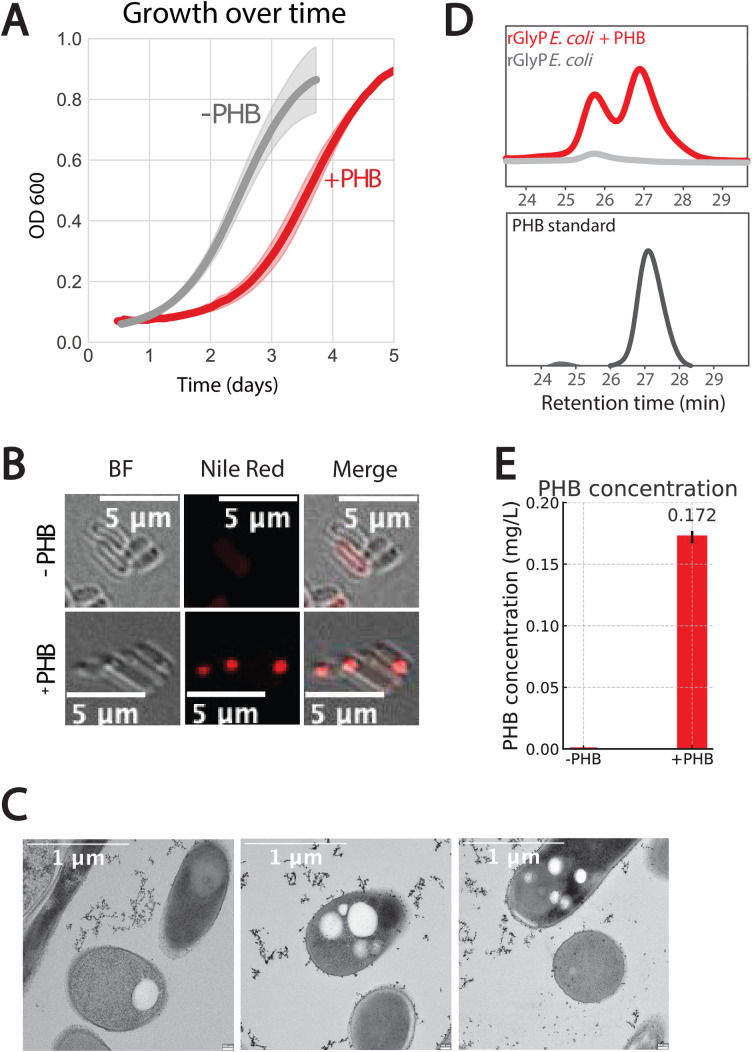
PHB production in formatotrophic E. coli engineered with the reductive glycine pathway. ***(A)*** *Growth curves of formatotrophic E. coli strains expressing the PHB operon (+PHB, red) or lacking it (−PHB, grey) in liquid M9 minimal media with 60 mM sodium formate and bubbled with a gas mixture of 10% CO*_*2*_*, 90% air (in duplicates). Cultures were grown in bioreactors and monitored for optical density at 600 nm (OD*_600_*).* ***(B)*** *Confocal fluorescence microscopy of Nile Red-stained cells. Brightfield (BF), Nile Red fluorescence, and merged images show intracellular PHB granules in the + PHB strain, absent in the − PHB control. Images were taken using a Nikon A1R HD25 confocal microscope, 60x. Images were cropped to show relevant fields of view and no post-processing was performed. Scale bars, 5 μm.* ***(C)*** *Transmission electron microscopy (TEM) of +PHB cells shows characteristic electron-transparent PHB granules (white inclusions) inside engineered E. coli. Scale bars, 1 μm.* ***(D)*** *HPLC chromatograms following sulfuric acid digestion of dried biomass. A crotonic acid peak at ~27 min is observed in the + PHB strain and matches the PHB standard. No such peak is detected in the control.* ***(E)*** *Quantification of PHB production by HPLC. The engineered strain accumulated 0.172 mg/L PHB, whereas the control strain showed no detectable production. Error bars represent standard deviation of biological replicates (n = 2).*

The formatotrophic strain carrying the PHB genes was cultivated in a fermenter using minimal media supplemented with 60 mM formate (as was previously determined to be the optimal concentration [18]) and an overlay of 10% CO_2_ in the headspace. The PHB-producing strain cultivated in the fermenter exhibited a prolonged lag phase compared to the non-PHB producing strain, suggesting that the synthesis of PHB slows bacterial growth (Fig 2A). This was expected, as it is well-established that heterologous production of chemicals or proteins tends to slow microbial growth [26].

To confirm PHB production, we first employed the widely-used staining technique using Nile red [27–29]. Nile red is a stain that becomes fluorescent within hydrophobic cell structures, enabling detection through fluorescent confocal microscopy. Using this, we observed the formation of granules (in red) in a transformed strain of *E. coli* (Fig 2B). In contrast, we did not observe such granules in a strain lacking the PHB-producing plasmid. As Nile Red also fluoresces when binding to bacterial membranes, we complemented these results with transmission electron microscopy, which reveals the morphology of the granules (Fig 2C). To quantify PHB production in formatotrophic *E. coli*, we performed acid-catalyzed depolymerization of harvested biomass, converting intracellular PHB into crotonic acid for detection by high-performance liquid chromatography (HPLC). The engineered strain expressing the PHB operon exhibited a distinct peak at 27 min, matching the retention time of a PHB-derived crotonic acid standard (Fig 1D). In contrast, no such peak was detected in the control strain lacking PHB biosynthesis genes. A second, smaller peak at ~25.75 min was also observed specifically in the engineered strain but not in the control, suggesting it may be a minor byproduct of PHB-related metabolism. As its identity remains unknown and does not interfere significantly with quantification, it was excluded from analysis. Quantification was performed using a PHB standard curve generated from acid-digested PHB standards (S3 Fig), revealing a PHB concentration of 0.172 ± 0.005 mg/L in the engineered strain, with no measurable PHB accumulation in the control (Fig 1E). These data confirm successful heterologous expression of the PHB pathway and demonstrate the strain’s ability to convert C1 substrates into a polymeric carbon storage compound under formatotrophic growth conditions.

## Discussion

It was previously demonstrated that *E. coli* can be engineered to grow on C1 compounds such as CO_2_, formate or methanol [18,19,21]. In this study, we use a synthetic formate-utilizing strain as a platform for the production of PHB. Through heterologous expression of the PHB producing pathway from *C. necator*, we successfully produced bio-plastic with a yield of 0.172 ± 0.005 mg/L of PHB after 120 hours of incubation. Although the achieved PHB yield is relatively low compared to traditional sugar-based processes—for example, recombinant *E. coli* strains have been shown to produce up to 6.21 g/L of PHB from sucrose in batch fermentation after 48 hours [30], it represents an important proof-of-concept for bioplastic production directly from C1 substrates. Key limitations of CO₂/formate-based systems include slow growth rates, gas transfer inefficiencies, and the high energy demands of CO₂ fixation. Future work should focus on improving strain performance, optimizing pathway expression, increasing carbon flux toward PHB, and refining cultivation conditions to enhance overall yield and process efficiency. Recent studies have begun to address some of these challenges by engineering faster-growing formatotrophic strains [31]. Integrating such improvements could enable more efficient and scalable PHB production from C1 feedstocks.

The gold standard in the field of C1 metabolism is to perform ^13^C labeling experiments to ensure that all the carbon is derived from CO_2_ and formate [18,19]. This method has already been used to demonstrate that the formatotrophic strain of *E. coli* synthesizes all of its biomass carbon exclusively from CO_2_ and formate in minimal media conditions. Since we used the identical strain described by Kim and collaborators, under identical growth conditions (in both the growth and in the production phases) we assumed that all the carbon used for PHB production came exclusively from CO_2_ and formate as well, as these are the only sources of carbon available in the growth medium.

### Decoupling and other strategies for improving the production capacities

A high PHB yield is critical for financially competing with traditional plastic production as this greatly influences the cost of production. Here we examine various strategies aimed at maximizing PHB yields, thereby advancing its potential for commercial viability. We also note that a pipeline for rapid quantification of PHB will be instrumental for progress in these efforts.

The production of PHB is a burden on cell growth. It is known that production of value-added chemicals also leads to the accumulation of toxins and mutations [32]. Expressing genes continuously requires a substantial amount of energy. It is more energy-efficient to express genes only when they are needed. To overcome these problems in bioproduction and reduce metabolic stress, it is common to decouple biomass accumulation from chemical production [33]. Current decoupling strategies include using inducible promoters triggered by physical (e.g., temperature) [34] or chemical (e.g. pH, nutrient depletion, IPTG and galactose) signals [35]. Recently, complex circuit designs for dynamic regulation, such as optogenetics [36] and biosensing [37], were shown to successfully increase the product yield in several bioproduction strains. An additional decoupling strategy for synthetic or natively autotrophic organisms is to use a two-stage cultivation system [15,38,39]. This consists of a heterotrophic growth phase to reach a high OD and an autotrophic growth phase for production. For formatotrophs, different strategies can be used to decouple growth from production including using formate inducible promoters [40]. A two-stage cultivation system is a more robust and reliable approach for sustainable bioproduction compared to constitutive production of a molecule of interest. Further investigation is warranted to explore the potential of a two-stage cultivation system using synthetic C1 feeding organisms.

Another approach to enhance PHB accumulation is by increasing the growth rate of the initial strains as it minimizes the fermentation costs and improves the overall robustness of the process. This can be accomplished through laboratory evolution or genetic modifications. Another viable strategy is modification of bacterial metabolic pathways to boost PHB production. Specific genetic manipulations include modulating phasin expression, which is a surface-binding protein of polyhydroxyalkanoate (PHA) granules that is encoded by the *phaP* gene. Indeed, an *E. coli* strain harboring novel phasins from a highly productive PHB bacterium, *Halomonas sp*. YLGW01, exhibited increased PHB production by around 3-fold. [41]. Additionally, the deletion of *ldhA, pta*, and *adhE* genes, which encode enzymes in competing metabolic pathways, can be employed to redirect resources toward PHB production. The composition of the growth medium can exert a significant impact on PHB accumulation.

Modifying the physical characteristics of bacterial cells can also positively influence PHB accumulation. Strategies may encompass altering cell size and shape, division patterns or cell wall structures [42]. For instance, by overexpressing *sulA*, a cell division inhibitor, in *E. coli* harboring PHB synthesis operon, engineered cells became long filaments and accumulated more PHB compared with the wild-type [43].

Finally, *In vitro* polymerization of PHB could be another way for overcoming spatial constraints within the cytoplasm. Traditional PHB production occurs inside bacterial cells, however, developing techniques for *in vitro* (outside the cell) polymerization of PHB has the potential to streamline the production process. This approach may offer greater control over the polymer’s characteristics and reduce energy and resource consumption.

### Potential for photovoltaic-driven bioproduction

Electron carriers generated through electrolysis, such as formate and hydrogen, are essential in photovoltaic-driven production to shuttle electrons to microorganisms that grow on C1 feedstocks.

Formate can be produced by electrochemical reduction of CO_2_ with a Faradaic efficiency greater than 90% [44,45]. Compared to hydrogen, the electrochemical production of formate is less efficient, but it has the advantage of being soluble and non-flammable, and thus more convenient for large-scale usage.

In the electrochemical reduction of CO_2_, only carbon monoxide and formate exhibit efficient production [46,47]. Since both require only a two-electron reduction of CO_2_, finding a suitable catalyst for their production is less challenging compared to multi-electron products such as methane, methanol, ethylene, oxalate, and acetate [48,47]. Exploring the bioproduction capabilities of organisms that use formate and CO_2_ as an energy and carbon source might therefore be a solution for achieving sustainable production of goods such as plastics.

Engineering an *E. coli* strain with rGlyP to heterologously express the PHB operon is a step in the long path towards electro-fermentation. To the best of our knowledge, this is the first report of bioproduction in a synthetic *E. coli* strain feeding on formate and CO_2_. This study serves as a proof of concept for synthetically engineering *E. coli* strains that can utilize formate and CO_2_ as a feedstock for the production of plastics and more generally the potential of photovoltaic-driven bioproduction for future sustainable production.

## Materials and methods

### General experimental design

The experimental work was designed to assess PHB production in engineered *E. coli* strains capable of utilizing CO₂ and formate as carbon sources via the reductive glycine pathway. Baseline cultivation conditions were adopted from Kim et al. (2020), who demonstrated robust growth of *E. coli* on formate and CO₂ via the rGlyP pathway. Across all experiments, key parameters such as temperature, gas composition, media formulation, and agitation were maintained constant. Potential factors influencing PHB production include formate concentration, CO₂ availability, and the physiological state of the culture at the time of sampling.

### Chemicals and reagents

Primers were synthesized by Sigma-Aldrich. PCR reactions were performed using KAPA HiFi HotStart ReadyMix or Taq Ready Mix. Glycine, Sodium formate, D-xylose, D-glucose, Nile-Red, PHB and 3HB standards were purchased from Sigma-Aldrich.

### Bacterial strains

The engineered *E. coli* strain used in this study was derived from the C1-assimilating strain described by Kim et al. (2020) [18]. The parental strain is based on *E. coli* K-12 MG1655 background, carrying chromosomally integrated modules enabling growth on formate and CO₂ via the reductive glycine pathway (rGlyP). The strain includes genetic modifications for formate assimilation, glycine/serine biosynthesis, and pathway optimization, as detailed by Kim et al. Further modifications to introduce the PHB biosynthetic pathway are described below. The strain has not been deposited in a public collection center.

### Recombinant plasmids

The plasmid used to introduce the PHB biosynthetic pathway was pHB-4, which was obtained from Kang Zhou (Addgene plasmid #140957; http://n2t.net/addgene:140957; RRID:Addgene_140957). This plasmid carries genes encoding the key enzymes required for PHB production.

### Bacterial cultivation

Growth methods were adapted from [18]. *E. coli* strains were initially propagated in LB medium at 37 °C with shaking at 250 rpm. Overnight LB cultures were used to inoculate a preculture at an OD_600_ of 0.02 in M9 minimal medium (50 mM Na₂HPO₄, 20 mM KH₂PO₄, 1 mM NaCl, 20 mM NH₄Cl, 2 mM MgSO₄, and 100 μM CaCl₂) supplemented with trace elements (134 μM EDTA, 13 μM FeCl₃·6H₂O, 6.2 μM ZnCl₂, 0.76 μM CuCl₂·2H₂O, 0.42 μM CoCl₂·2H₂O, 1.62 μM H₃BO₃, and 0.081 μM MnCl₂·4H₂O), 10 mM glucose, 1 mM glycine, and 30 mM sodium formate. Precultures were grown at 37 °C with shaking at 250 rpm in 10-mL glass tubes under a 10% CO₂/ 90% air atmosphere. Cells were collected by centrifugation (18,407g, 3 min, 4 °C), washed twice with fresh M9 medium to remove residual carbon sources, and inoculated into M9 minimal medium supplemented with 30 mM or 60 mM sodium formate. Cultures were incubated at 37 °C with shaking at 250 rpm under 10% CO₂/ 90% air for 120 hours to allow full adaptation to formate. After 120 hours, cells were collected by centrifugation and used to inoculate the main cultures for either growth analysis or PHB production experiments. For strains carrying the PHB biosynthetic plasmid, 25 μg/mL chloramphenicol was included in all cultivation steps following preculture to maintain plasmid selection. For growth curve experiments, adapted cells were transferred into a DASBox multi-parallel fermentation system (Eppendorf) containing 140 mL M9 medium supplemented with 60 mM sodium formate. Cultures were inoculated to an initial OD_600_ of ~0.05 and grown at 37 °C with constant agitation under a 10% CO₂/ 90% air gas mixture. Growth was continuously monitored, and when cultures reached saturation, they were diluted back to the starting OD_600_ by removing culture and replenishing with fresh sterile medium. For PHB quantification experiments, adapted cells were inoculated to an initial OD_600_ of ~0.005 into 500 mL baffled Erlenmeyer flasks containing 250 of M9 minimal medium supplemented with 30–60 mM sodium formate (with chloramphenicol for plasmid strains). Cultures were incubated at 37 °C with shaking at 250 rpm under a 10% CO₂/ 90% air atmosphere for 120 hours to accumulate sufficient biomass for PHB extraction and HPLC analysis.

### Nile Red confocal microscopy

Nile red is a fluorescent dye used to visualize lipid-like inclusions. This dye binds to PHB granules and can be detected by fluorescence microscopy. Nile red staining was performed according to the protocol detailed in [27]. Cells were harvested by centrifugation (6,000x). 4 μl of cells were taken and pipet mixed with 1 μl of Nile red (10 μg/ml in DMSO). Then, 1 μl of the stained cell suspension was put on a microscopic slide and covered with an agarose pad. The cells were imaged using a Nikon A1R HD25 confocal microscope. Nile Red fluorescence was detected with DU4 detector, excitation of 561.5 nm laser, and emission filter 593/46 nm. Fluorescence intensity was adjusted to the negative control and imaged with the SR Plan Apo IR AC 60x water immersion objective (numerical aperture 1.27). Images were cropped to show relevant fields of view.

### PHB quantification by HPLC

PHB content was quantified following acid digestion and HPLC analysis. Briefly, 250 mL of cell culture was collected, centrifuged at maximum speed for 20–30 minutes at room temperature, and the pellet was dried at 90°C overnight. Dried pellets were digested by adding 0.5 mL concentrated sulfuric acid (99.999%, Sigma-Aldrich, catalog #339741) and incubating at 90°C for 30 minutes in a heat block, with vortexing every 10 minutes. After digestion, samples were cooled at room temperature for 30 minutes, diluted with 0.5 mL of 5 mM H₂SO₄, vortexed, filtered (0.22 μm syringe filter), and transferred to HPLC vials

PHB standards (0.005–1 mg) were prepared by dissolving poly[(R)-3-hydroxybutyric acid] (Sigma-Aldrich) in chloroform, aliquoting the appropriate volumes into glass vials, and allowing the solvent to evaporate overnight. The resulting dry PHB films were digested using concentrated sulfuric acid, following the same procedure as for the cell biomass samples.

Samples were analyzed using a Bio-Rad Aminex HPX-87H column at 40°C with a mobile phase of 5 mM H₂SO₄ at a flow rate of 0.6 mL/min. The injection volume was 30 μL. A photodiode array (PDA) detector collected signals between 190–300 nm, and quantification was performed at 210 nm. The main PHB degradation product, crotonic acid, eluted at approximately 27 minutes under these conditions.

HPLC analysis of PHB *E. coli* samples revealed two distinct peaks: one corresponding to crotonic acid eluted at ~27 minutes, and an unknown entity eluted at ~25.75 minutes. To account for this peak overlap, the crotonic acid peak area was determined as the area under the 27-minute peak to the right of a vertical line drawn at the lowest point in the detector signal between the 25.75-minute and 27-minute peaks, extending down to the x-axis. This integration approach likely underestimates the actual area of the 27-minute peak, as it would be larger if the 25.75-minute peak were absent.

### Electron microscopy

Samples for electron microscopy were routinely collected along the experiments and cells were harvested. Cells were then placed in an aluminum disc with a depression of 100 μm and outer diameter of 3 mm (Engineering Office M. Wohlwend GmbH), then covered with a matching flat disc. The sandwiched sample was high‐pressure frozen using an EM ICE high pressure-freezing device (Leica Microsystems, GmbH, Germany). Frozen samples were dehydrated in a temperature-controlled AFS2 Freeze substitution device (Leica Microsystems). Substitution was performed in dry acetone containing 1% glutaraldehyde, 1% osmium tetroxide and 0.1% Uranyl Acetate at -90 °C for 64 h. The temperature was gradually increased to -20 °C (2.9 °C/h) and then raised to 4° C (12 °C/h). The samples were washed five times with acetone and infiltrated for 4 days at room temperature in a series of increasing concentrations of Epon in acetone. After polymerization at 60 °C for 48 h, ultrathin sections (90 nm) were obtained using an EMUC7 ultramicrotome (Leica microsystems) and were mounted on formvar coated 200 mesh nickel grids. Sections were stained with Reynolds lead citrate and examined using a Thermo Fisher Scientific Tecnai T12 transmission electron microscope operating at 120 kV. Digital electron micrographs were acquired using a bottom mounted TVIPS TemCam-XF416 4k x 4k CMOS camera.

## Supporting information

S1 Fig HPLC chromatograms confirming PHB production in formatotrophic E. coli. (A) Chromatograms of acid-digested biomass from two biological replicates of rGlyP E. coli expressing the PHB operon (+PHB). Crotonic acid peaks are consistently observed at 27 min. (B) Corresponding chromatograms from control strains lacking PHB operon show no detectable crotonic acid signal. (C) Chromatogram of the PHB standard digested under identical conditions confirms peak identity and retention time. Detection was performed at 210 nm using a photodiode array detector.
(TIF)

S2 Fig Continuous OD600 measurements of rGlyP E. coli and rGlyP + PHB E. coli in liquid M9 minimal media with 60 mM sodium formate and sparged with a gas mixture of 10% CO_2_, 90% air. Growth was carried out in the DASBox mini fermentation system (150 mL working volume). The time axes are different.
(TIF)

S3 FigStandard curve used for PHB quantification by HPLC (log-log regression).A standard curve was generated using digested PHB standards at 0.002, 0.008, 0.053, 0.055, and 0.5 mg/mL. The resulting crotonic acid peak areas (detected at 210 nm) were plotted against PHB concentration. Due to skew caused by clustering of low-concentration data, a log-log transformation was applied. Power-law regression yielded the equation: $Area=1.14\times 10^{8}\cdot {Conc}^{0.88},\;R^{2}=0.993$ This model more accurately captures the nonlinear relationship across several orders of magnitude and was used to quantify PHB content in experimental samples following acid digestion and HPLC analysis.(TIF)
