# Supplementary figures and images for: Demonstration of bioplastic production from CO2 and formate using the reductive glycine pathway in E. coli
Source: PLoS One. 2025 Jul 22;20(7):e0327512. doi: 10.1371/journal.pone.0327512 (PMC12282915; doi:10.1371/journal.pone.0327512)

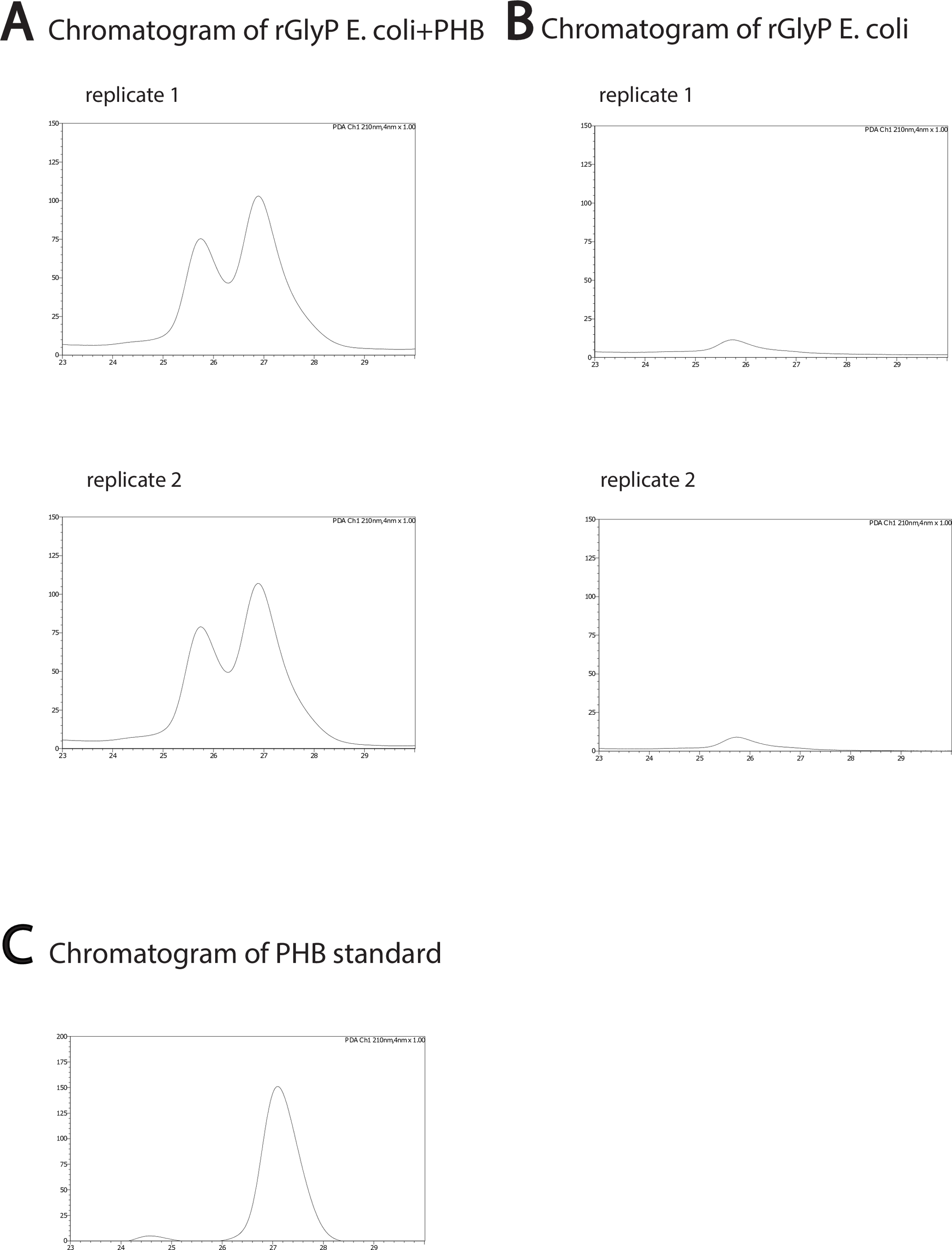

Supplement: S1 Fig — (A) Chromatograms of acid-digested biomass from two biological replicates of rGlyP E. coli expressing the PHB operon (+PHB). Crotonic acid peaks are consistently observed at 27 min. (B) Corresponding chromatograms from control strains lacking PHB operon show no detectable crotonic acid signal. (C) Chromatogram of the PHB standard digested under identical conditions confirms peak identity and retention time. Detection was performed at 210 nm using a photodiode array detector. (TIF) [file pone.0327512.s001.tif]

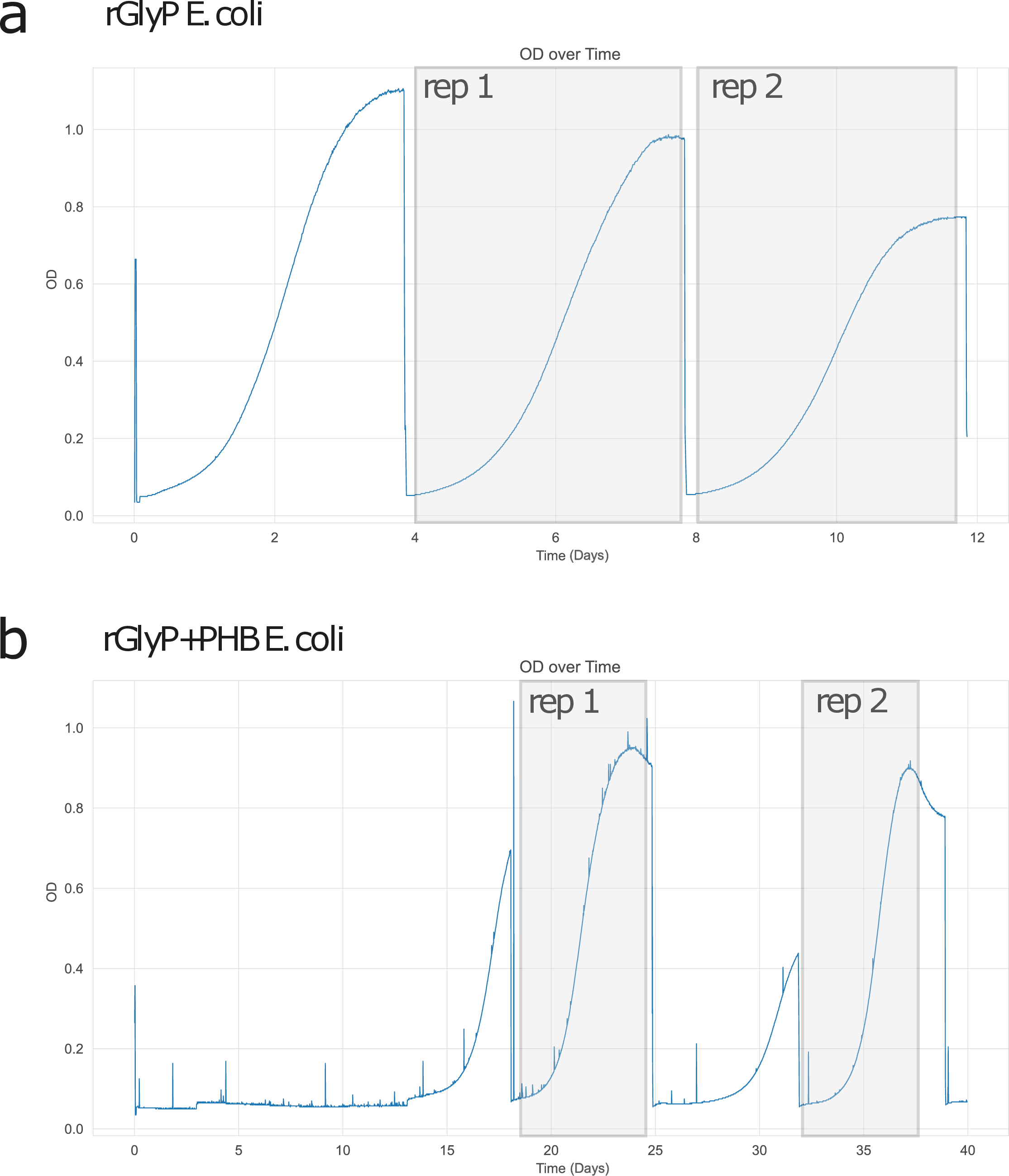

Supplement: S2 Fig — Growth was carried out in the DASBox mini fermentation system (150 mL working volume). The time axes are different. (TIF) [file pone.0327512.s002.tif]

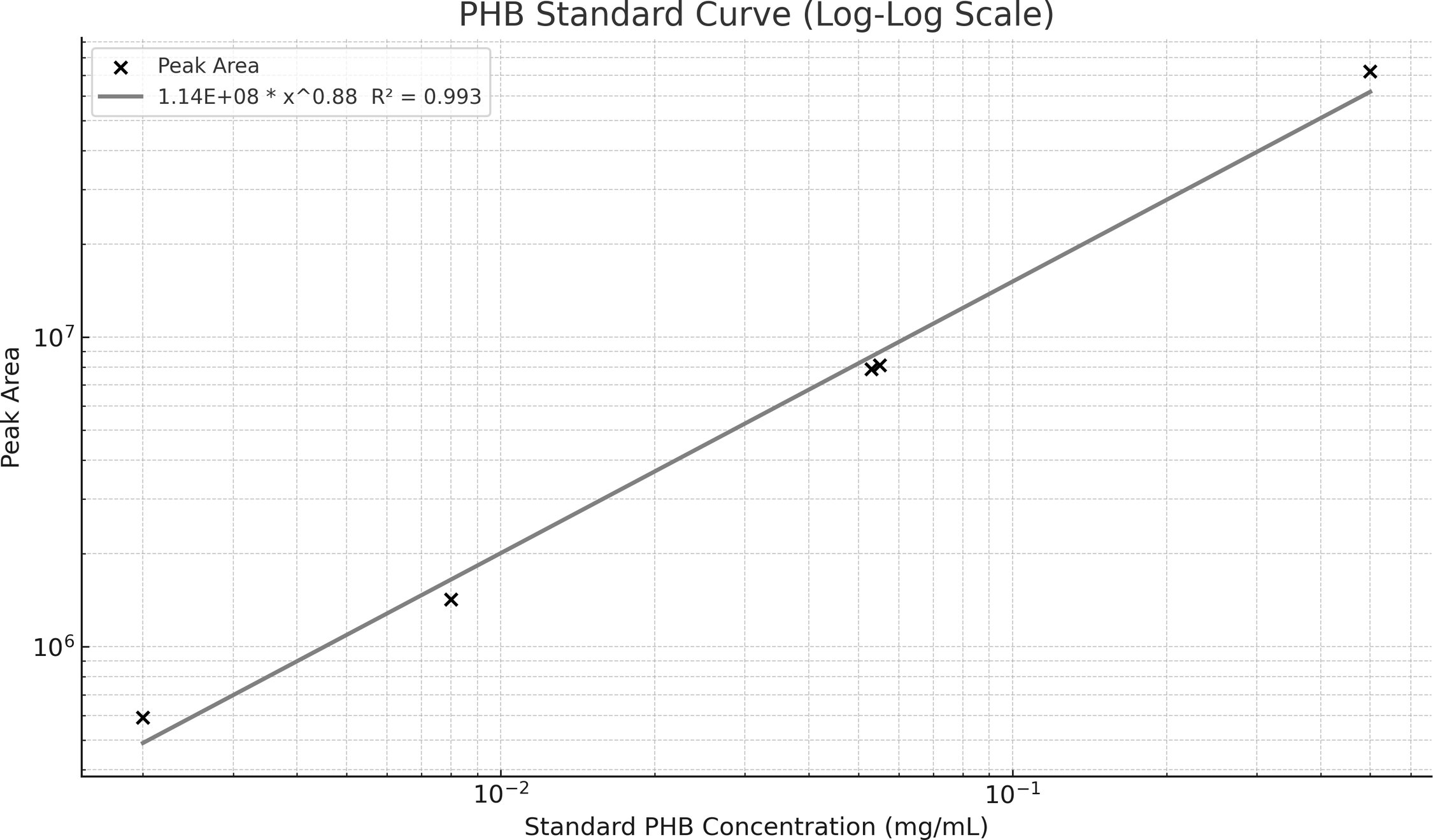

Supplement: S3 Fig — A standard curve was generated using digested PHB standards at 0.002, 0.008, 0.053, 0.055, and 0.5 mg/mL. The resulting crotonic acid peak areas (detected at 210 nm) were plotted against PHB concentration. Due to skew caused by clustering of low-concentration data, a log-log transformation was applied. Power-law regression yielded the equation: Area=1.14×10^8^·Conc0.88, R2=0.993 This model more accurately captures the nonlinear relationship across several orders of magnitude and was used to quantify PHB content in experimental samples following acid digestion and HPLC analysis. (TIF) [file pone.0327512.s003.tif]
